# Pesquisa em sepse pediátrica em países de baixa e média renda: superando desafios

**DOI:** 10.5935/0103-507X.20210062

**Published:** 2021

**Authors:** Daniela Carla de Souza, Cláudio Flauzino de Oliveira, Vanessa Soares Lanziotti

**Affiliations:** 1 Unidade de Terapia Intensiva Pediátrica, Hospital Universitário, Universidade de São Paulo - São Paulo (SP), Brasil.; 2 Instituto Latino Americano de Sepse - São Paulo (SP), Brasil.; 3 Unidade de Terapia Intensiva Pediátrica, Divisão de Pesquisa e Ensino, Programa de Pós-Graduação em Saúde Materno-Infantil, Universidade Federal do Rio de Janeiro - Rio de Janeiro (RJ), Brasil.

## INTRODUCTION

### The burden of pediatric sepsis

Sepsis represent one of the most frequent acute medical conditions in the intensive care units, and one of the main causes of morbidity and mortality in children.^(1)^ Most of the deaths occur in low- and middle-income countries (LMIC), particularly in Sub-Saharian Africa and south Asia, and are due to preventable diseases.^(2, 3)^ Data published in 2020, derived from the 2017 Global Burden of Disease, estimated 48.9 million cases of sepsis and 11 million deaths, representing 19.7% of deaths of all causes of death. More than one-half of the cases of sepsis (25.2 million) involved children and adolescents, with a total of 3.3 million deaths.^(4)^ A systematic review including 15 studies from 12 countries - most of them developed countries - reported a prevalence of sepsis and severe sepsis in children of 48 cases and 22 cases/100,000 people-year, corresponding to a 9% and 22% mortality, respectively.^(5)^ Another systematic review and metanalysis, that included 94 studies and 7,561 pediatric patients with sepsis and organ dysfunctions, showed a decreasing trend in lethality for the period between 1980 and 2016, however, has also shown that the mortality remains high (25%) and is larger in LMIC: the chance of a child with sepsis dying in developing countries is four times higher than in developed countries.^(6)^

We still do not fully know the impact of pediatric sepsis. The scarce data in the literature is believed to be underestimated, especially in LMIC. In light of this, in 2017 the World Health Organization (WHO) recognized sepsis as a global health problem and started demanding the member countries measures for prevention, diagnosis, and treatment.^(7)^ However, in LMIC these measures will only be effectively implemented if we can know pediatric sepsis impact in these regions, by conducting clinical and epidemiological studies.

### Challenges for pediatric sepsis research in low- and middle-income countries

Clinical research is always challenging, even more, when involving severely ill children and, especially, in LMIC, where resources are limited. Children are historically excluded from clinical trials, mainly for the sake of ethical issues. New drugs and procedures are generally used in pediatric patients by extrapolation of data from adult clinical trials.^(8)^ According to the PICUtrials website (http://picutrials.net/), which registers pediatric intensive care unit (PICU) randomized controlled clinical trials (RCTs), from 1986 to January 2021 483 RCTs were registered, from which only 33 (6.8%) involved sepsis in children.^(9)^ Most of the trials were from developed countries, involving one or two sites, and including between 50 and 100 children.

Less than one out 100 admissions to the PICUs are recruited to clinical trials.^(10)^ The inclusion of children is more difficult due to aspects such as vulnerability and, mainly in LMIC, ethical issues involving parental informed consent, given the possible cultural gap between researchers and participants, many of them with poor schooling and difficulty in understanding.

Another issue for sepsis trials is related to the syndrome characteristics: variable and nonspecific presentation, complex pathophysiology, dynamics, and heterogeneous group of patients.^(11)^ In the pediatric population this variability and heterogeneity are more evident, considering the different age groups, rapidly changing clinical presentation and hemodynamics, and lower mortality rate in comparison with the adult population, factors that make it difficult to conduct research.^(12)^

In limited-resources settings, clinical practice is generally not based on strong evidences, but rather on expert opinion or experience, and information from studies conducted in more economically advanced countries. This “adapted” approach to LMIC can be a huge misunderstanding, as shown by the FEAST trial,^(13)^ that raised concern on aggressive fluid resuscitation in septic shock children in limited-resources settings, and led to a review of the septic shock treatment guidelines, which can be seen in the new recommendations of the Surviving Sepsis Campaign.^(14)^

**Figure 1:**
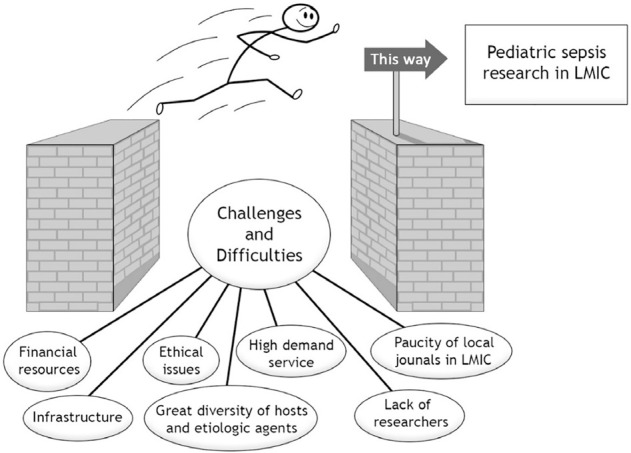
Challenges and difficulties related to research in low- and middle-income countries. LMIC - low- and middle-income countries

Despite the relevance and little knowledge of pediatric sepsis in LMIC, few resources are invested in this research, resulting in deficient infrastructure, overloaded care demand, little incentive to train new researchers, and the lack of resources specifically allocated for research (Figure 1). The disparity in the use of financial resources for research in the different regions of the world is shown by the “10-90 gap”: only 10% of the resources destined for health research in the world are directed to pathologies that affect 90% of the poorest population.^(15)^ Based on data from the World Bank, Argent et al. reported that the availability of healthcare resources can vary up to 100 times in different regions of the planet, and up to 10 times among LMIC.^(16)^

Shortage of experient researchers, lack of a clinical research culture, and work overload in the PICU are mentioned as barriers for researching in LMIC, as mentioned by the PALISI Global Health.^(17)^ Other limitations include lack of research training, difficulty recruiting patients, lack of time exclusively dedicated to research, lack of a research career, difficulty with statistical support, different risk prediction/stratification models, limited capacity of microbiology services, the unpreparedness of ethics boards, inexperience in writing manuscripts, and difficulty publishing in impact journals.^(17, 18)^ The shortage of local scientific journals makes it even more difficult to encourage research and publish the unique reality of these countries, impacting not only the amount but also the quality of clinical trials.

Other challenges involve a diversity of hosts, etiological agents, and scenarios, in particular in the settings of evident economical and infrastructure inequalities.^(19^-^21)^ The pediatric sepsis spectrum in LMIC is even bigger, with a larger populational heterogeneity (host) and a broader spectrum of agents, including tuberculosis, arboviruses, protozoans, and other parasites, uncommon in developed countries.^(20)^

### It is possible conducting clinical trials under adverse conditions

Despite difficulties, LMIC should invest in research and production of their own evidence, since we do not know enough about the impact of sepsis and we need to reduce risk and morbidity in these regions. According to Argent et al., in the LMIC the pediatric sepsis approach, including research, should be pragmatic and not ideal, to mitigate the shortage of resources.^(16)^

Researchers from these regions have been performing good quality RCTs, changing paradigms. In Brazil, de Oliveira et al. have shown that reanimation of children with septic shock according to the American College of Critical Care Medicine/Pediatric Advanced Life Support (ACCM/PALS) guidelines may result in lower morbidity and mortality, when associated with continuous monitoring of the central venous oxygen saturation (SvcO_2_). Goal-directed resuscitation, aiming at SvcO_2_ ≥ 70%, had a significant impact on the outcome of children with septic shock.^(22)^ Also in Brazil, Ventura et al. have shown that the use of dopamine as the first choice drug in pediatric septic shock was associated with an increased risk of death and infection. Early peripheral or intraosseous epinephrine significantly increased survival.^(23)^

In India, Ranjit et al., through a multimodal assessment, gained a better understanding of the hemodynamic status in fluid-refractory septic shock, demonstrating that the hyperdynamic profile is also common in pediatric sepsis, offering a rationale for the early use of norepinephrine.^(24)^ The same authors assessed fluid-restrictive resuscitation in children with septic shock (30mL/kg) and early norepinephrine, concluding that this strategy may be beneficial as compared with the ACCM guidelines, as the patients in the intervention group received fewer fluids and had more mechanical ventilation-free days.^(25)^ Studies like these promoted changes to international diagnosis and therapy guidelines, showing that LMIC are capable of producing their own evidences, in addition to having a larger number of cases. However, these are still one-off initiatives, the result of great individual efforts.

### Perspectives in pediatric sepsis research in low- and middle-income countries

Some solutions can foster research in developing countries. Duffet et al. pointed out some interventions to promote conducting RCTs in PICUs: collaborating and working with experienced researchers and international research networks, strengthening the culture of clinical research as a quality improvement process in institutions, and seeking financial resources in developing countries through research collaboration and funding agencies. Cultural changes related to the importance of clinical trials in building evidence to improve care practice and support of departments and institutions are also primary steps.^(18)^

Collaboration between low/middle- and high-income countries provides the possibility of formal training for conducting research, writing and publishing manuscripts, protocols, and guidelines. Some initiatives such as the Methods in Epidemiologic, Clinical, and Operations Research Program (MECOR), a research training program in LMIC developed by the American Thoracic Society (ATS), have been training young researchers around the world.^(26)^

### The importance of multicenter studies, large databases, and collaborative networks

According to the SPROUT trial, considering the PICU mortality rates (the most used outcome in clinical trials), it is estimated that 1,059 patients per group (control/intervention), in 58 PICUs for three years would be required to access a 5% reduction of the risk of death (80% power); this confirms that well-designed mortality outcome trials will be only feasible with collaboration and multicenter trials.^(1)^

In the last years, relevant PICU trials have been published based on data from large databanks.^(27^-^29)^ Although there are structural and procedural differences among hospital institutions, large systematically recording databanks can adjust index-cases and help to understand the clinical practice. Additionally, they can provide feasibility data for RCTs, monitor performance, and provide strategic planning for the improvement of several diseases’ prognoses.

Collaborative networks can facilitate performing RCTs, both quantitative and qualitatively, providing advances in several diseases’ knowledge and prognosis. According to Choong et al., a research network can be defined as a formal, collective, cooperative, or collaborative consortium, aimed at facilitating the conduction of clinical trials.^(10)^ Networks help to identify and prioritize research agendas, establishing common interest subjects and promoting experient investigators-led research. Studies conducted by research networks can involve larger numbers of subjects and assess more relevant outcomes, with better quality evidences and greater chances of publication in impact journals, and a higher number of citations. Also, have better chances of receiving funding from development agencies.

In developed countries, some networks as the Canadian Critical Care Trials Group (Canada), Pediatric Acute Lung Injury and Sepsis Investigators - PALISI (USA) e Australian and New Zealand Intensive Care Society Clinical Trials Group - ANZICS-CTG (Australia and New Zealand) are examples of well-established pediatric intensive care groups. However, the number of RCTs from these networks is still relatively small and so far has not included any pediatric sepsis trial.

In the last years, collaborative research networks in pediatric intensive care are being developed in LMIC. Specifically in Latin America, two collaborative research networks were created: Red Colaborativa Pediátrica Latinoamérica (LaRED), with the mission of improving the safety of medical care for children and their families by a coordinated program of research, education, and quality improvement; and Brazilian Research Network in Pediatric Intensive Care (BRnet PIC) aimed at helping PICUs and researchers in conduction of clinical, translational, and epidemiologic trials.

## Final considerations

Considering that the vast majority of deaths in children under the age of five years occur in limited-resource countries due to infectious diseases and sepsis, research in these regions must become a priority, providing production of local evidence, and the development of specific guidelines to reduce morbidity and mortality in children with sepsis. Available evidence about pediatric sepsis in LMIC suggests that the treatment of these patients is not being carried out in the best way. These findings highlight that LMIC must produce their own evidences, ceasing to be only coadjuvants and users of evidences produced by high-income countries. This is a challenging, but a necessary path.
